# Ciguatera Fish Poisoning in East Asia and Southeast Asia

**DOI:** 10.3390/md13063466

**Published:** 2015-06-02

**Authors:** Thomas Y. K. Chan

**Affiliations:** 1 Division of Clinical Pharmacology and Drug and Poisons Information Bureau, Department of Medicine and Therapeutics, Faculty of Medicine, The Chinese University of Hong Kong, Prince of Wales Hospital, Shatin, New Territories, Hong Kong, China; E-Mail: tykchan@cuhk.edu.hk; Tel.: +852-2632-3907; Fax: +852-2646-8756; 2 Centre for Food and Drug Safety, Faculty of Medicine, The Chinese University of Hong Kong, Hong Kong, China

**Keywords:** ciguatera, ciguatoxins, East Asia, Southeast Asia

## Abstract

In the coastal countries of East Asia and Southeast Asia, ciguatera should be common because of the extensive tropical and subtropical coral reefs along the coasts and in the neighboring seas with ciguatoxic fishes. An extensive search of journal databases, the Internet and the government websites was performed to identify all reports of ciguatera from the regions. Based on the official data and large published case series, the incidence of ciguatera was higher in the coastal cities (Hong Kong, Foshan, Zhongshan) of southern China than in Japan (Okinawa Prefecture). In Singapore, ciguatera appeared to be almost unknown. In other countries, only isolated cases or small case series were reported, but under-reporting was assumed to be common. Ciguatera may cause severe acute illness and prolonged neurological symptoms. Ciguatera represents an important public health issue for endemic regions, with significant socio-economic impact. Coordinated strategies to improve risk assessment, risk management and risk communication are required. The systematic collection of accurate data on the incidence and epidemiology of ciguatera should enable better assessment and management of its risk. Much more work needs to be done to define the size threshold for important coral reef fish species from different regions, above which the risk of ciguatera significantly increases.

## 1. Introduction

Ciguatera is caused by consumption of tropical and subtropical reef fishes that have accumulated ciguatoxins (CTX) [1,2]. CTX precursors are produced by the dinoflagellates *Gambierdiscus* species. Large predatory fishes (e.g., moray eels, Spanish mackerels, groupers, barracuda and snappers) account for most of the reported cases [2,3,4]. Although ciguatoxic fishes and large outbreaks are mainly found in discrete regions of the Pacific Ocean, Indian Ocean and Caribbean Sea, between the latitudes 35°N and 35°S, the incidence and geographical distribution of ciguatera are increasing because of increased fish trade and consumption, international tourism and climate changes [2,5]. CTX found in the Pacific (P-CTX), Caribbean (C-CTX) and Indian Ocean (I-CTX) regions differ in toxicity (P-CTX > I-CTX > C-CTX) [1]. This reflects the regional differences in the composition of *Gambierdiscus* species, which show a >100-fold variation in toxicity [6]. Ciguatera is characterized by gastrointestinal, neurological, cardiovascular and other features [1,2,3,4]. The severity and occurrence of symptoms may reflect the amounts and types of CTX involved and ingestion of CTX-rich fish parts (head, viscera, roe and skin) and concomitant consumption of alcohol may cause more severe poisoning and prolonged illness [3,4,7,8].

**Figure 1:**
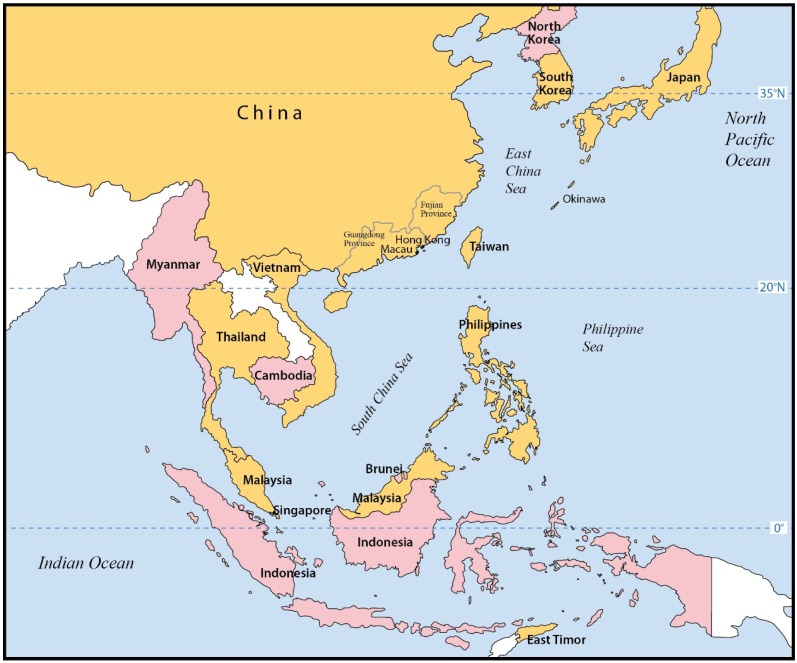
Reports of Ciguatera in the Coastal Countries of East Asia and Southeast Asia. 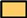 Countries with ≥1 report identified; 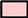 Countries with 0 report identified.

In the Pacific Ocean region, the changing incidence and geographical distribution of ciguatera are relatively well defined only in the Pacific Islands and Australia [9,10,11]. In the present review, the main objective is to report the incidence and epidemiology of ciguatera in the coastal countries of East Asia and Southeast Asia (Figure 1), where there are rich supplies and growing demand for coral reef fishes.

## 2. Reports of Ciguatera

To identify journal articles from the coastal countries of East Asia (China including Hong Kong and Macau, Japan, South Korea, North Korea and Taiwan) and Southeast Asia (Brunei, Cambodia, East Timor, Indonesia, Malaysia, Myanmar, Philippines, Singapore, Thailand and Vietnam), a search of the Medline (1980 to 26 February 2015), China Journal Jet (1994 to February 2015) and KoreaMed was performed, using ciguatera and ciguatoxins as the keywords. To identify non-indexed journal articles, conference reports and other relevant publications available in the Internet, a search of Google Scholar and Google was performed, using ciguatera, ciguatoxins and the country names as the search terms. In addition, the government websites were searched for official reports and press releases on ciguatera.

An extensive search of journal databases, the Internet and the government websites did not identify any reports of ciguatera in Brunei, Cambodia, Indonesia, Myanmar and North Korea. Only countries with at least one report of ciguatera would be considered further: China including Hong Kong and Macau, East Timor, Japan, Malaysia, Philippines, Singapore, South Korea, Taiwan, Thailand and Vietnam.

The epidemiology of ciguatera in China [12] and Hong Kong [13] and the outbreaks caused by tiger grouper [3] and humphead wrasse [4] were recently reviewed. Since the epidemiological features were already described in detail [3,4,12,13], only a summary was given below and in Table 1.

**Table 1:** Epidemiology of ciguatera in China and Hong Kong.

	China [12] 1994–2008	Hong Kong [13] 1989–2008
Geographical distribution of reports	Guangdong Province (92%)	Territory-wide
Peak in number of reports or cases	1994 (58% of reports)	2 peaks in cases (1998, 2004)
Incidence per million people (year)	1.1 (2005/6) ^a^ to 7.5 (2004) ^a^	3.3 to 64.9 (median 10.2)
	>48.7 (2004) ^b^	1st peak—64.9 (1998)
	>129.9 (2004) ^c^	2nd peak—35.5 (2004)
Large outbreaks (>100–200 subjects) ^d^	3	0
Important fish species ^e^	Tiger grouper, humphead	Snappers (until 1996)
	wrasse, areolated coral grouper	Groupers (from 1997) ^e^

Based on city-wide figures in ^a^ Shenzhen and hospital-based case series in ^b^ Foshan and ^c^ Zhongshan. ^d^ Caused by tiger grouper served at the banquets. ^e^ Groupers (tiger grouper, leopard coral grouper, lyretail grouper, flowery grouper, spotted coral grouper), moray eel, two-spot red snapper and humphead wrasse, *etc.*, were commonly involved.

In China, there were altogether 24 reports of ciguatera in 1994–2008. In Dalian, Liaoning Province, 1 outbreak involving 2 subjects was caused by humphead wrasse [14]. In Xiamen, Fujian Province, 3 outbreaks involving 29 subjects were caused by tiger grouper [15]. In Guangdong Province [12], there were 22 reports (including 3 large outbreaks each affecting over 100–200 subjects after eating tiger grouper served at the banquets): Dongguan (*n* = 1), Foshan (*n* = 5), Guangzhou (*n* = 1), Shantou (*n* = 4), Shenzhen (*n* = 5), Yangjiang (*n* = 1), Zhongshan (*n* = 3) and Zhuhai (*n* = 2). One other report of 2 exported cases to Macau in 2004 (2 adults sharing a humphead wrasse >2.4 kg in Shenzhen) was identified [16]. In Hong Kong, there were 3–117 outbreaks (median 19), affecting 19–425 subjects (median 68) each year in 1989–2008, with an annual incidence of 3.3–64.9 (median 10.2) per million people [13].

Table 2 summarizes all reports of ciguatera in the region, other than China and Hong Kong. Since the clinical features of ciguatera in the Pacific [1,12,13] are well recognized, only the epidemiological features and possible risk factors were highlighted here. News media reports would only be mentioned.

In Macau, according to the Centre for Disease Control and Prevention, there were 2 outbreaks in 2005–2006 involving 12 subjects [17,18], in addition to 2 imported cases from Shenzhen in 2004 [16].

In East Timor, there was an informal report from Oxfam; a Dili-based staff member was diagnosed with ciguatera [19].

In Japan, until the 1980s, ciguatera was restricted to the subtropical areas—Okinawa Prefecture and Amami Islands. In Ryukyu and Amani Islands, according to the organizations concerned with public health or fisheries and other sources, there were 99 outbreaks up to 1968, affecting ~477 subjects [20]. The incidence appeared to have increased since over two-thirds of the outbreaks occurred after 1950. In 1949–1980, there were 23 outbreaks affecting 379 subjects [21]. Since the 1990s, ciguatera also occurred in the temperate regions. In 1989–2010, the Ministry of Health, Labour and Welfare reported 78 outbreaks affecting 284 subjects [22]; ciguatera occurred predominately in Okinawa (89.7%) and Kagoshima (3.8%), but cases were reported from prefectures further north (6.4%). Local reports from Okinawa in 1997–2006 [23] and 2008 [24] and from Amami Islands in 2006 [25], which were covered in the nationwide report [22], would be used for estimation of annual incidence only, if applicable. In contrast, local reports completely or incompletely left out were listed in Table 2. In 1999, 1 outbreak in Chiba affected 10 subjects [26]. In 2005–2008, 6 outbreaks (3 outbreaks in nationwide report [22]) in Kagoshima involved 13 subjects, with an annual incidence of 2.2 per 100,000 people in the Kakeroma Island [27]. In 2008, 1 outbreak in Mie affected 3 subjects [28].

In Malaysia, according to the 2 speakers of an annual local health conference in 2011, ciguatera was first reported in April 2010 (6 subjects in 3 clusters) [29,30]. In May 2010, 26 workers were involved, and in September 2010, 22 members from 5 families were involved [30]. All of these outbreaks were caused by red snapper.

In Philippines, ciguatera occurred in Basilan Province in August 1988, involving 19 subjects from 4 families, after eating a single barracuda [31]. Data from 60 foodborne disease outbreaks in 1995–2004 and health advice released in Department of Health website had been reviewed and 38 ciguatera cases were identified [32]. A poison center in France reported a case of imported ciguatera from Philippines [33]. In June 2010, 22 subjects from 2 families in Iloilo developed ciguatera after eating red snapper [34]. Not shown in Table 2 were local news reports cited by Mendoza, *et al.* [34]; there were 2 reports of ciguatera caused by barracuda in 2001 (50 subjects affected) and 2006 (33 subjects affected) and 4 reports of unknown types of fish poisoning in 2004–2008.

In Singapore, there was 1 report in April 2000 involving 2 subjects, caused by imported mackerel [35].

In South Korea, there were 2 reports, each involving 1 subject caused by imported cod or unknown fish [36,37].

In Taiwan, 11 outbreaks of ciguatera, each involving 1–5 subjects, occurred between June 1991 and December 2008 [38,39,40,41]. The causative species identified included moray eel *G. javanicus* (April 2004), red grouper *L. bohar* (February 2006, December 2008). In addition, there were two outbreaks caused by ingestion of barracuda eggs or viscera [42,43]. There was one death from ciguatera in 1998 after ingestion of red grouper [38].

**Table 2:** All reports of ciguatera in East Asia and Southeast Asia, other than China and Hong Kong.

Location [Ref.]	Period	Sex	Age (year)	Details
Macau				
[17]	November 2005	3MF ^a^	-	1 outbreak, after sharing a grouper ^e^ (>1.2 kg), all 3 subjects hospitalized
[18]	September 2006	9MF ^a^	-	1 outbreak, after sharing a grouper ^e^ (>1.8 kg), 3 subjects hospitalized
East Timor				
Dili [19]	2000	1MF ^a^	-	1 staff member of Oxfam developed symptoms while in East Timor ^e^, diagnosis made in Darwin
Japan				
Ryukyu & Amami Islands [20]	–1930 to 1968	~477MF ^a^	-	99 outbreaks ^g^, after eating *L. bohar* (39.4%), *E. fuscoguttatus* (12.1%), *G. flavimarginatus* (10.1%), *V. louti* (9.1%), *S. fuscescens* (9.1%), *Serranus* species (6.1%), *L. monostigma* (4.0%), *Cheilinus* species (3.0%), *Carcharhinus* species (2.0%) and other fishes, including fish head (26.3% outbreaks), viscera (5.1%) and skin (3.0%), >two-thirds of outbreaks occurred in 1951–1968, more common in summer
Nationwide [21]	1949 to 1980	379MF ^a^	-	23 outbreaks ^g^
Nationwide [22]	1989 to 2010	284MF ^a^	-	78 outbreaks ^g^, after eating *V. louti* (20.5%), *L. monostigma* (15.4%), *L. bohar* (14.1%), *O. punctatus* (7.7%) and other fishes, occurring in Okinawa (89.7%), Kagoshima (3.8%), Miyazaki (1.3%), Hyōgo (1.3%), Osaka (1.3%), Kanagawa (1.3%) and Ibaraki (1.3%) Prefectures
Chiba [26]	August 1999	10MF ^a^	-	1 outbreak in Katsuura, after eating *O. punctatus* ^f^
Kagoshima [27]	2005 to 2008	7M6F	38 (6–78) ^b^	6 outbreaks in Kakeroma Island, after eating *V. louti* (50%) and other fishes ^f,g^, annual incidence 0.2 per million population or 0.1 outbreak per million population
Mie [28]	July 2008	3MF ^a^	-	1 outbreak ^g^
Malaysia				
[29]	April 2010	6MF ^a^	-	3 outbreaks, after eating red snapper ^e^
[30]	May 2010	26MF ^a^	-	26 workers, after eating red snapper ^e^
Kelantan [30]	September 2010	12M10F	27 ^b^	5 families in Jeli, after eating red snapper ^f^ including fish head (36.3%) and viscera (13.6%), 11 subjects hospitalized
Philippines				
Basilan [31]	August 1988	8M11F	26 (4–61) ^c^	4 families, after eating parts of a single barracuda (*Sphyraena jello*) ^f^ caught in the vicinity of Basilan Island, all 19 subjects hospitalized, incidence in Isabela (the capital) was 0.4/100,000
Philippines (*cont.*) ^c^				
Nationwide? [32]	1995–2004	38MF ^a^	-	Data ^g^ from Field Epidemiology Training Program and advisories released by Department of Health
[33]	1997–2002	1MF ^a^	-	1 imported case of ciguatera ^e^ from the Philippines with treatment in southern France
Iloilo [34]	June 2010	22MF ^a^	(1–50)	2 families each consumed ~2 kg of red snapper (*Lutjanus campechanus*) ^e^, all 22 subjects hospitalized
Singapore				
[35]	April 2000	2MF ^a^	-	2 subjects, after eating imported mackerel ^e^ (Ministry of Health report)
South Korea				
Seoul [36]	February 2006	1M	56	1 subject hospitalized with coma and respiratory failure, after eating imported cod intestine ^e^
Jeonju [37]	-	1M	25	1 subject hospitalized, after eating some raw fish ^e^
Taiwan				
Province-wide [38,39,40,41]	1991–2008	26MF ^a^	-	11 outbreaks, after eating red grouper ^e,f^ (64%), toothed jobfish ^e^ (18%) ^e^, doctor fish ^e^ (9%) and moray eel ^e^ (9%), occurring in South (73%), North (18%) and West (9%) Taiwan, 1 death (red grouper)
Kaohsiung [42]	-	5MF ^a^	-	1 family, after eating barracuda eggs ^e^, 3 subjects hospitalized
Kaohsiung [43]	-	2M2F	(25–59)	1 family, after eating barracuda viscera ^e^, 1 subject (F/45) developed reversible corpus callosum lesion
Thailand				
[44]	January 1984	1F	29	1 imported case of ciguatera ^e^ from Thailand with hospitalization in Italy
Bangkok [45]	August 2007	2F	(20–50)	1 outbreak, after eating sea bass ^e^, all 2 subjects hospitalized (1 subject with respiratory failure)
Phuket [45]	December 2009	2M2F	(9–34)	1 family, after eating red snapper ^e^, all 4 subjects hospitalized
Vietnam ^d^				
[46]	-	1M	44	1 imported case of ciguatera ^e^ from Vietnam with medical treatment in Spain
Ninh Thuan & Binh Thuan [47]	May–June 2008	97MF ^a^	-	After eating (red) snapper ^f^
Binh Thuan [47]	2009–2013	~30MF ^a^	-	After eating (red) snapper ^f^
Quang Ngai [47]	August 2010	5MF ^a^	-	After eating (red) snapper ^f^

^a^ Total number of M and F; ^b^ Median or ^c^ mean age (range); ^c^ Local news reports of 2 outbreaks caused by barracuda (2001, 2006) and 4 outbreaks of unknown fish poisoning (2004–2008) not shown; ^d^ A news media report of 17 other cases caused by barracuda not shown; Fish tested for CTX—^e^ no, ^f^ yes, ^g^ no details.

In Thailand, an Italian tourist developed ciguatera after eating some marine fish [44]. During 2007–2009, there were 2 outbreaks affecting 2–4 subjects after ingestion of sea bass or red snapper [45].

In Vietnam, a Spanish tourist developed ciguatera after eating some fish [46]. According to the talk delivered at a scientific symposium organized by the IOC Sub-Commission for the Western Pacific, in 2008–2013, there were several outbreaks in Ninh Thuan, Binh Thuan and Quang Ngai, affecting ~132 subjects [47]. (Red) snapper was involved. Not shown in Table 2 was a news media report of 17 other cases caused by barracuda.

In the reports reviewed here, ciguatera was clinically diagnosed, but the criteria used were rarely described [30,31,34,45]. Where the fish remnants were available for testing, the presence of CTX was confirmed using mouse bioassay [23,24,25,27,38,39,40,41,47], LC–MS [23,25,30,47] or other methods [26,31,34]. In other reports (Table 2), fish remnants were not analyzed or there was no information about laboratory confirmation.

The annual incidence of ciguatera could be estimated in Okinawa Prefecture of Japan. In 1997–2006, there were 33 outbreaks affecting 103 subjects [23]. The October population size was provided in the official website of Okinawa Prefecture [48]. The overall annual incidence was 7.7 per million population. In 2008, 9 subjects were involved in 3 outbreaks [24], with an annual incidence of 6.5 per million population.

Possible risk factors for an increased likelihood of (severe) ciguatera [1,3,4,12,13], such as the consumption of CTX-rich fish parts (head, viscera, roe and skin), were observed in some of the outbreaks in Japan [20], Malaysia [30], South Korea [36] and Taiwan [42,43]. However, any concomitant use of alcohol and the fish size involved were not mentioned.

## 3. Discussion

Ciguatera may cause severe acute illness, including life-threatening bradycardia and hypotension [7], respiratory failure [45], coma, neuropsychiatric features [1,2] and, rarely, death [38]. Many individuals may suffer prolonged neurological illness, including fatigue, muscle weakness and paresthesia in the 4 limbs [1,2,8,49]. Thus, ciguatera, particularly its severe form [3], represents an important public health issue for endemic regions, with significant socio-economic impact, especially in developing territories [50]. To reduce the impact of ciguatera worldwide, coordinated strategies to improve risk assessment, risk management and risk communication are required [51]. The systematic collection of accurate data on the incidence and epidemiology of ciguatera should enable better assessment and management of its risk [52]. However, in the Pacific Ocean region, the changing incidence and geographical distribution of ciguatera are relatively well defined only in the Pacific Islands and Australia [9,10,11].

In the coastal countries of East Asia and Southeast Asia (Figure 1), ciguatera should be common as well for several reasons. There are extensive tropical and subtropical coral reefs along the coasts and in the neighboring seas [53] with ciguatoxic fishes (Table 1 and Table 2). Fish is a staple diet; certain fish species are food delicacies to local populations or commercially important to individual communities [20,22,31,40]. Over the past 2–3 decades, there is a remarkable growth in the demand for live coral reef fishes [12,13] to the extent that supplies are increasingly sought from other (new) fishing grounds [54]. Outbreaks can be caused by both local and imported reef fish [29,30,34]. For countries primarily relying on food imports, the major risk comes from the imported fish [55]. Climate changes [5] and coral reefs disruption (caused by increased human activities [53] and cyclones) may increase the incidence of ciguatera by favoring the growth and geographical distribution of the *Gambierdiscus* species [2,56].

In Hong Kong [13] and other coastal cities of southern China [12], the incidence and epidemiology of ciguatera were recently analyzed based on government figures and published case series in journals, respectively (Table 1). Official data are preferred if food poisoning (including ciguatera) is a notifiable disease and annual reports and periodical updates are published or accessible via government websites. As for published case series, emerging importance and large outbreaks will favor reporting [12].

Similarly, in Singapore and Japan, the incidence of ciguatera could be estimated, using the official statistics (Table 2). In Singapore, ciguatera appeared to be almost unknown [55], since only 2 cases in 2000 could be identified [35] by communicable disease surveillance from 1993 to 2014. In Japan, until the 1980s, ciguatera reports were restricted to the subtropical regions (Okinawa Prefecture and Amami Islands) [20]. Since the 1990s, ciguatera also occurred in the temperate areas [22]. During 1989–2010, there were 78 outbreaks affecting 284 subjects, occurring predominately in Okinawa (89.7%) and Kagoshima (3.8%) but less commonly in prefectures further north (6.4%) [22]. In Okinawa Prefecture, 103 subjects were involved in 33 outbreaks during 1997–2006 [23] (overall annual incidence 7.7 per million population). In 2008, 3 outbreaks affected 9 subjects (annual incidence 6.5 per million population). In Kakeroma Island, Kagoshima Prefecture, 13 subjects were involved in 6 outbreaks during 2005–2006 [27] (annual incidence 0.2 per million population). Only isolated cases or small case series were reported from other countries and territories (Table 2), which may be partly attributable to under-reporting [30,34].

It appears that the incidence of ciguatera was much higher in the coastal cities of southern China than in Japan (Table 1 and Table 2). In Foshan and Zhongshan, because of large outbreaks affecting >100–200 subjects related to *E. fuscoguttatus* served at banquets, the incidence was the highest in 2004 (>48.7 and >129.9 per million population). In Hong Kong, the incidence was the highest in 1998 (64.9 per million population), after reef fishes were imported from a new fishing ground [54].

The predominant reef fish species responsible for ciguatera outbreaks differ across countries (Table 1 and Table 2), depending on the ciguatoxic potential of local and imported fish species. The geographical origins of the fish species (with regional variations in ciguatoxic potential) and the local preference for the fish types and size as food delicacy are important factors [3,4].

Ciguatera is generally a clinical diagnosis based on the characteristic signs and symptoms occurring shortly after eating reef fish that are known to contain CTX [1,2,3,4]. Recent advances in methodologies (LC–MS) provide not only confirmatory test, but also CTX quantification in reef fish [51]. In addition, species-specific and region-specific CTX profiles [57,58], the Pacific origin of ciguatoxic fish [57,58,59] and P-CTX-1 dominating the CTX profiles [59] are confirmed. Exposure to P-CTX-1 (the most potent CTX) may explain the predominance of acute cardiovascular features (bradycardia and hypotension) and neurological symptoms in affected subjects [3,4,12,13].

For risk assessment and preventive measures, it would be useful to know the size of fish involved in ciguatera outbreaks. In Hong Kong, over 80% of the reef fish associated with ciguatera outbreaks were >2 kg [60]. Thus, avoiding eating fish >2 kg is a reasonable approach, but occasionally fish weighing 0.6 kg or less are also implicated [13]. It should be remembered that the prevalence of ciguatoxicity is also species- [3,4] and, in particular, region-specific [23,61].

Much more work needs to be done to define the size (length) threshold, for important fish species from different regions (Table 2), above which the risk of ciguatera significantly increases [23]. For example, in Okinawa, Japan, *L. bohar* <4 kg were non-toxic, but the prevalence of toxicity (mouse bioassay ≥0.025 MU/g) rose to 37.7% in fish >4 kg and 61.1% in fish >7 kg [23]. In New Caledonian, *L. bohar* was considered ciguatoxic, whatever its size [61]. In French Polynesia, there was a lack of relationship between toxicity and size for most of the fish species and families [62].

Concomitant alcohol consumption may be associated with more severe ciguatera illness [3,4]. Such information was missing in previous reports (Table 2). The available data did indicate that consumption of CTX-rich fish parts was common (Table 2), confirming the continuing need for public education, especially for communities in the endemic regions.

The public health impact of ciguatera is underestimated because of under-reporting. The reluctance to report illness may reflect the lack of conviction that anything can be done for ciguatera [50]. Non-reporting of cases can still be common even if ciguatera is a recognizable disease [63]. As mild cases may be mistaken for more common illnesses [50,63], the diagnosis may be missed and affected individuals may not seek medical attention.

## 4. Conclusions

In the coastal countries of East Asia and Southeast Asia, there are extensive tropical and subtropical coral reefs along the coasts and in the neighboring seas with ciguatoxic fishes. Thus, ciguatera should be common. Official figures and large published case series were available for estimation of incidence in Hong Kong and other coastal cities of southern China, Singapore and Japan. Only isolated cases or small case series were reported from other countries. Ciguatera can cause both severe acute illness and prolonged neurological symptoms. Ciguatera represents an important public health issue for endemic areas, with significant socio-economic impact. Coordinated strategies to improve risk assessment, risk management and risk communication are required. The systematic collection of accurate data on the incidence and epidemiology of ciguatera should enable better assessment and management of its risk.
